# Mental health literacy and its associated factors among undergraduate students: preliminary findings from SMILE Project

**DOI:** 10.1192/j.eurpsy.2025.2017

**Published:** 2025-08-26

**Authors:** A. I. Teixeira, S. Lima, V. Pereira, F. Pinto, T. Morgado, S. Martins

**Affiliations:** 1 Health Sciences, Nursing School of Tâmega e Sousa IPSN-CESPU; 2 Innovation in Health and Well-Being – Research Unit, Instituto Politécnico de Saúde do Norte, CESPU; 3 Coimbra College of Nursing and the Leiria College of Health; UICISA:E and RISE@CINTESIS, Penafiel, Portugal

## Abstract

**Introduction:**

Mental health literacy (MHL) is crucial for undergraduate students as it empowers them to recognize, understand, and address mental health challenges. This knowledge contributes to improved happiness by reducing stigma, fostering resilience, and promoting positive coping strategies. Enhanced emotional well-being results from effective stress management and the ability to seek help when needed. Ultimately, mental health literacy positively influences academic life satisfaction by creating a supportive environment, reducing the impact of stressors, and fostering a holistic approach to well-being in the challenging context of higher education.

**Objectives:**

To assess the association between MHL and satisfaction with academic life, happiness, psychological well-being and psychiatric symptoms in undergraduate students. It also aimed to analyse the relationship between MHL and sociodemographic and clinical characteristics of these students.

**Methods:**

Under the ongoing prospective project called “SMILE”, this preliminary study integrated a sample of undergraduate students from a private higher education institution in northern of Portugal. The research protocol included a semi-structured interview (sociodemographic and clinical data) and the Mental Health Literacy Questionnaire (MHLq-SVa), Satisfaction with Academic Life Scale (SALS), Subjective Happiness Scale (SHS), Psychological Well-Being Scale (PWBS) and Depression Anxiety Stress Scale (DASS). Non-parametric statistical tests were used, as the data did not follow a normal distribution.

**Results:**

Twenty-four students were evaluated (75% female, 92% single; mean age=25.5y y.o.). Around 67% were nursing students and 42% in their 4^th^ year. Regarding support needs for mental health problems, 74% had support at some point in their lives and about 17% were currently receiving support. (22% were taking psychotropic medications). Also, 57% had a family history of mental disorders. Women had a greater knowledge of mental health problems (MHI domain), compared to men (median=28vs.25; p=0.025). This MHL domain was negatively correlated with satisfaction with the academic environment (rs= -0,571). Higher scores in MHL total and in its self-help strategies domain were identified among students with previous mental health support needs (median=72vs69.5, p=0.20; median=19vs18, p=0.44, respectively). Lower level of erroneous beliefs/stereotypes (MHI domain) were more evident in students with family mental health history (median=15vs13.5, p=0.007). No relations were found between MHL and other assessed variables.

**Conclusions:**

These findings are important for designing interventions to promote mental health in academic contexts, aimed at positive and healthy changes in the academic environment, particularly in line with their expectations and needs, with the aim of reducing levels of psychological distress.

**Disclosure of Interest:**

None Declared

